# 1-year health outcomes associated with systemic corticosteroids for COVID-19: a longitudinal cohort study

**DOI:** 10.1183/23120541.00474-2024

**Published:** 2024-09-30

**Authors:** Olivia C. Leavy, Richard J. Russell, Ewen M. Harrison, Nazir I. Lone, Steven Kerr, Annemarie B. Docherty, Aziz Sheikh, Matthew Richardson, Omer Elneima, Neil J. Greening, Victoria Claire Harris, Linzy Houchen-Wolloff, Hamish J.C. McAuley, Ruth M. Saunders, Marco Sereno, Aarti Shikotra, Amisha Singapuri, Raminder Aul, Paul Beirne, Charlotte E. Bolton, Jeremy S. Brown, Gourab Choudhury, Nawar Diar Bakerly, Nicholas Easom, Carlos Echevarria, Jonathan Fuld, Nick Hart, John R. Hurst, Mark Jones, Dhruv Parekh, Paul Pfeffer, Najib M. Rahman, Sarah Rowland-Jones, Ajay M. Shah, Dan G. Wootton, Caroline Jolley, A.A. Roger Thompson, Trudie Chalder, Melanie J. Davies, Anthony De Soyza, John R. Geddes, William Greenhalf, Simon Heller, Luke Howard, Joseph Jacob, R. Gisli Jenkins, Janet M. Lord, Will D-C. Man, Gerry P. McCann, Stefan Neubauer, Peter J.M. Openshaw, Joanna Porter, Matthew J. Rowland, Janet T. Scott, Malcolm G. Semple, Sally J. Singh, David Thomas, Mark Toshner, Keir Lewis, Liam G. Heaney, Andrew Briggs, Bang Zheng, Mathew Thorpe, Jennifer K. Quint, James D. Chalmers, Ling-Pei Ho, Alex Horsley, Michael Marks, Krisnah Poinasamy, Betty Raman, Louise V. Wain, Christopher E. Brightling, Rachael A. Evans

**Affiliations:** 1 Department of Population Health Sciences, University of Leicester, Leicester, UK; 2 The Institute for Lung Health, NIHR Leicester Biomedical Research Centre, University of Leicester, Leicester, UK; 3 Centre for Medical Informatics, The Usher Institute, University of Edinburgh, Edinburgh, UK; 4 The Usher Institute, University of Edinburgh, Edinburgh, UK; 5 Royal Infirmary of Edinburgh, NHS Lothian, Edinburgh, UK; 6 Roslin Institute, University of Edinburgh, Edinburgh, UK; 7 University Hospitals of Leicester NHS Trust, Leicester, UK; 8 Centre for Exercise and Rehabilitation Science, NIHR Leicester Biomedical Research Centre-Respiratory, University of Leicester, Leicester, UK; 9 Department of Respiratory Sciences, University of Leicester, Leicester, UK; 10 Therapy Department, University Hospitals of Leicester, NHS Trust, Leicester, UK; 11 NIHR Leicester Biomedical Research Centre, University of Leicester, Leicester, UK; 12 St George's University Hospitals NHS Foundation Trust, London, UK; 13 The Leeds Teaching Hospitals NHS Trust, Leeds, UK; 14 University of Nottingham, Nottingham, UK; 15 Nottingham University Hospitals NHS Trust, Nottingham, UK; 16 NIHR Nottingham Biomedical Research Centre, Nottingham, UK; 17 UCL Respiratory, Department of Medicine, University College London, Rayne Institute, London, UK; 18 University of Edinburgh, Edinburgh, UK; 19 NHS Lothian, Edinburgh, UK; 20 Manchester Metropolitan University, Manchester, UK; 21 Salford Royal NHS Foundation Trust, Manchester, UK; 22 Infection Research Group, Hull University Teaching Hospitals, Hull, UK; 23 University of Hull, Hull, UK; 24 The Newcastle Upon Tyne Hospitals NHS Foundation Trust, Newcastle Upon Tyne, UK; 25 Translational and Clinical Research Institute, Newcastle University, Newcastle Upon Tyne, UK; 26 Department of Respiratory Medicine, Cambridge University Hospitals NHS Foundation Trust, Cambridge, UK; 27 University of Cambridge, Cambridge, UK; 28 NIHR Cambridge Clinical Research Facility, Cambridge, UK; 29 Lane Fox Respiratory Service, Guy's and St Thomas NHS Foundation Trust, London, UK; 30 University College London, London, UK; 31 Royal Free London NHS Foundation Trust, London, UK; 32 Clinical and Experimental Sciences, Faculty of Medicine, University of Southampton, Southampton, UK; 33 NIHR Southampton Biomedical Research Centre, University Hospitals Southampton, Southampton, UK; 34 University of Birmingham, Birmingham, UK; 35 University Hospital Birmingham NHS Foundation Trust, Birmingham, UK; 36 Barts Health NHS Trust, London, UK; 37 Queen Mary University of London, London, UK; 38 Oxford University Hospitals NHS Foundation Trust, Oxford, UK; 39 University of Oxford, Oxford, UK; 40 NIHR Oxford Biomedical Research Centre, Oxford, UK; 41 CAMS Oxford Institute, Oxford, UK; 42 University of Sheffield, Sheffield, UK; 43 Sheffield Teaching NHS Foundation Trust, Sheffield, UK; 44 Kings College London, London, UK; 45 Kings College London NHS Foundation Trust, London, UK; 46 Institute of Infection, Veterinary and Ecological Sciences, University of Liverpool, Liverpool, UK; 47 Liverpool University Hospitals NHS Foundation Trust, Liverpool, UK; 48 NIHR Health Protection Research Unit in Emerging and Zoonotic Infections, University of Liverpool, Liverpool, UK; 49 Department of Psychological Medicine, Institute of Psychiatry, Psychology and Neuroscience, Kings College London, London, UK; 50 South London and Maudsley NHS Trust, London, UK; 51 Diabetes Research Centre, University of Leicester, Leicester, UK; 52 Population Health Sciences Institute, Newcastle University, Newcastle Upon Tyne, UK; 53 Newcastle upon Tyne Teaching Hospitals Trust, Newcastle upon Tyne, UK; 54 NIHR Oxford Health Biomedical Research Centre, University of Oxford, Oxford, UK; 55 Oxford Health NHS Foundation Trust, Oxford, UK; 56 University of Liverpool, Liverpool, UK; 57 The CRUK Liverpool Experimental Cancer Medicine Centre, Liverpool, UK; 58 Department of Oncology and Metabolism, University of Sheffield, Sheffield, UK; 59 Imperial College Healthcare NHS Trust, London, UK; 60 Imperial College London, London, UK; 61 Centre for Medical Image Computing, University College London, London, UK; 62 Lungs for Living Research Centre, University College London, London, UK; 63 National Heart and Lung Institute, Imperial College London, London, UK; 64 MRC Versus Arthritis Centre for Musculoskeletal Ageing Research, Institute of Inflammation and Ageing, University of Birmingham, Birmingham, UK; 65 NIHR Birmingham Biomedical Research Centre, University Hospitals Birmingham and the University of Birmingham, Birmingham, UK; 66 Royal Brompton and Harefield Clinical Group, Guy's and St Thomas NHS Foundation Trust, London, UK; 67 NHLI, Imperial College London, London, UK; 68 Department of Cardiovascular Sciences, University of Leicester, Leicester, UK; 69 Division of Cardiovascular Medicine, Radcliffe Department of Medicine, University of Oxford, Oxford, UK; 70 ILD Service, University College London Hospital, London, UK; 71 Kadoorie Centre for Critical Care Research, Nuffield Department of Clinical Neurosciences, University of Oxford, Oxford, UK; 72 MRC–University of Glasgow Centre for Virus Research, Glasgow, UK; 73 NIHR Health Protection Research Unit in Emerging and Zoonotic Infections, Institute of Infection, Veterinary and Ecological Sciences, University of Liverpool, Liverpool, UK; 74 Respiratory Medicine, Alder Hey Children's Hospital, Liverpool, UK; 75 Cambridge NIHR BRC, Cambridge, UK; 76 Hywel Dda University Health Board, Wales, UK; 77 University of Swansea, Wales, UK; 78 Respiratory Innovation Wales, Wales, UK; 79 Wellcome–Wolfson Institute for Experimental Medicine, Queen's University Belfast, Belfast, UK; 80 Belfast Health and Social Care Trust, Belfast, UK; 81 London School of Hygiene and Tropical Medicine, London, UK; 82 University of Dundee, Ninewells Hospital and Medical School, Dundee, UK; 83 MRC Human Immunology Unit, University of Oxford, Oxford, UK; 84 Division of Infection, Immunity and Respiratory Medicine, Faculty of Biology, Medicine and Health, University of Manchester, Manchester, UK; 85 Manchester University NHS Foundation Trust, Manchester, UK; 86 Department of Clinical Research, London School of Hygiene and Tropical Medicine, London, UK; 87 Hospital for Tropical Diseases, University College London Hospital, London, UK; 88 Division of Infection and Immunity, University College London, London, UK; 89 Asthma and Lung UK, London, UK; 90 Radcliffe Department of Medicine, University of Oxford, Oxford, UK; 91 These authors contributed equally

## Abstract

**Background:**

In patients with coronavirus disease 2019 (COVID-19) requiring supplemental oxygen, dexamethasone reduces acute severity and improves survival, but longer-term effects are unknown. We hypothesised that systemic corticosteroid administration during acute COVID-19 would be associated with improved health-related quality of life (HRQoL) 1 year after discharge.

**Methods:**

Adults admitted to hospital between February 2020 and March 2021 for COVID-19 and meeting current guideline recommendations for dexamethasone treatment were included using two prospective UK cohort studies (Post-hospitalisation COVID-19 and the International Severe Acute Respiratory and emerging Infection Consortium). HRQoL, assessed by the EuroQol-Five Dimensions–Five Levels utility index (EQ-5D-5L UI), pre-hospital and 1 year after discharge were compared between those receiving corticosteroids or not after propensity weighting for treatment. Secondary outcomes included patient-reported recovery, physical and mental health status, and measures of organ impairment. Sensitivity analyses were undertaken to account for survival and selection bias.

**Findings:**

Of the 1888 participants included in the primary analysis, 1149 received corticosteroids. There was no between-group difference in EQ-5D-5L UI at 1 year (mean difference 0.004, 95% CI −0.026–0.034). A similar reduction in EQ-5D-5L UI was seen at 1 year between corticosteroid exposed and nonexposed groups (mean±sd change −0.12±0.22 *versus* −0.11±0.22). Overall, there were no differences in secondary outcome measures. After sensitivity analyses modelled using a cohort of 109 318 patients admitted to hospital with COVID-19, EQ-5D-5L UI at 1 year remained similar between the two groups.

**Interpretation:**

Systemic corticosteroids for acute COVID-19 have no impact on the large reduction in HRQoL 1 year after hospital discharge. Treatments to address the persistent reduction in HRQoL are urgently needed.

## Shareable abstract

**Systemic corticosteroids given for acute COVID-19 do not affect health-related quality of life or other patient-reported outcomes, physical and mental health outcomes, or organ function 1 year after hospital discharge.** https://bit.ly/3XR45Ln

## Introduction

The discovery of vaccines and effective treatments for acute coronavirus disease 2019 (COVID-19) (corticosteroids (dexamethasone), anti-interleukin (IL)-6 agents, monoclonal antibodies and Janus kinase inhibitors) have reduced progression to invasive mechanical ventilation and improved mortality [1–4]. However, many survivors experience persistent symptoms, physical and mental health effects, cognitive impairment, and multi-system organ damage, which can reduce health-related quality of life (HRQoL) for years after the initial infection (at least 4 years to date) [5–7].

Definitions for post-COVID-19 sequelae vary [8, 9], but the patient-derived term “long COVID” is now commonly used to describe persistent symptoms beyond 4 weeks after the acute infection [10]. The mechanisms underlying long COVID are complex, multifaceted and not yet fully understood, but potentially include persistent inflammation, which is associated with the severity of ongoing health impairments [5, 11]. Corticosteroids prescribed for acute COVID-19 requiring supplemental oxygen may potentially reduce the risk and severity of long COVID by attenuating the acute inflammatory burden [12].

Many of the large acute COVID-19 therapeutic trials, including RECOVERY [1–4], did not have detailed follow-up, which limits understanding of the longer-term effects, and it would now be unethical to randomise patients to placebo rather than corticosteroids. Adults previously randomised to receive acute corticosteroids on intensive care showed no improvement in HRQoL at 6 months compared to usual care [13], although a small observational study suggested a modest benefit in some quality of life domains and persistence of symptoms in patients who had received corticosteroids [12]. We have previously reported no acute corticosteroid effect on patient-perceived recovery at 1 year [6]. However, it is unknown whether corticosteroids during acute COVID-19 requiring supplemental oxygen affect other longer-term sequelae.

Using data from the PHOSP-COVID (Post-hospitalisation COVID-19) [14] and ISARIC (International Severe Acute Respiratory and emerging Infection Collaboration) [15] studies, we aimed to investigate whether treatment with corticosteroids in patients with COVID-19 requiring oxygen supplementation was associated with improved HRQoL 1 year after hospital discharge. Additionally, we aimed to investigate the effect of acute corticosteroids on a broad range of secondary health outcomes.

## Methods

### Study design

This was a longitudinal cohort study using data from two UK multicentre prospective cohort studies. Adults discharged from hospital after COVID-19 between 1 February 2020 and 31 March 2021 were recruited from 36 UK National Health Service (NHS) hospital sites as part of the PHOSP-COVID study previously described [14]. Data were collected 1 year after hospital discharge, including patient-reported recovery, physical and mental health status, and measures of organ impairment (detailed below). Pre-hospital EuroQol-Five Dimensions–Five Levels utility index (EQ-5D-5L UI) was completed retrospectively at a study visit 2–7 months after hospital discharge, with participants considering their quality of life prior to admission for COVID-19.

For the sensitivity analysis, we used data from the ISARIC study [15], which included more than 300 000 patients admitted to over 200 NHS hospitals across England, Scotland and Wales with COVID-19.

### Participants

**FIGURE 1:**
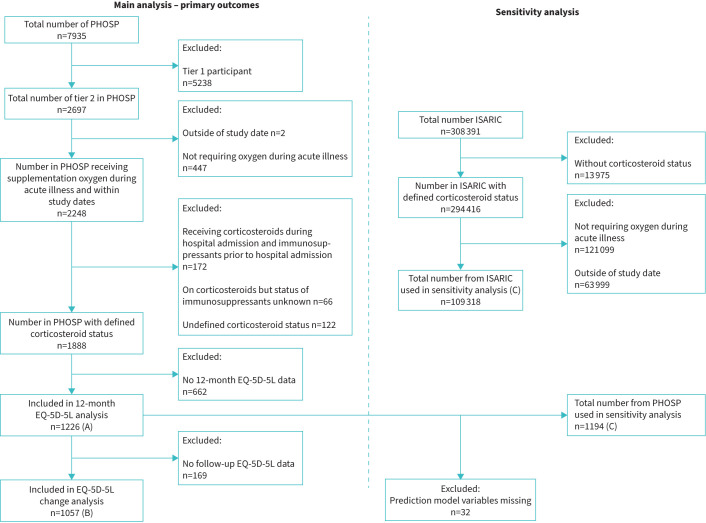
Consort diagram demonstrating study population included in co-primary outcomes of the EuroQol-Five Dimensions–Five Levels utility index (EQ-5D-5L UI) at 1 year (A), and change in EQ-5D-5L UI from pre-hospital to 1 year (B), and sensitivity analysis (C). “Tier 1” participants had collection of routine clinical data with linkage to retrospective and prospective health and social care records only. “Tier 2” participants underwent enhanced clinical data collection and research-specific biosampling at two further research visits following hospital discharge, including collection of the study outcomes. ISARIC: International Severe Acute Respiratory and emerging Infection Consortium cohort; PHOSP: Post-hospitalisation cohort.

Eligibility criteria for PHOSP-COVID have been previously described in detail [14]. For this analysis we selected participants who required supplemental oxygen therapy (World Health Organization (WHO) clinical progression scale 5), noninvasive ventilatory support (WHO clinical progression scale 6) or invasive mechanical ventilation (WHO clinical progression scale 7–9) [16] during their hospital admission in accordance with current guideline requirements for corticosteroid use in COVID-19 [17] and who had completed an EQ-5D-5L UI at their 1 year study visit. We excluded patients on pre-existing immunosuppressant medications (including systemic corticosteroids in the 14 days prior to hospital admission) and where corticosteroid exposure was unknown or not recorded (figure 1).

For the sensitivity analysis, we analysed a subset of the ISARIC study cohort, who were admitted with COVID-19 in the same study period and meeting the same WHO clinical progression scale criteria [15] (figure 1).

### Exposure

Patients who received any systemic (oral or intravenous) corticosteroid during their hospital admission for COVID-19 were compared to those who did not.

### Outcomes

The primary outcome was HRQoL, assessed by the EQ-5D-5L UI [18]. EQ-5D-5L UI 1 year after hospital discharge and change in EQ-5D-5L UI from pre-hospital to 1 year were compared between corticosteroid exposed and nonexposed patients.

Secondary outcomes were patient-perceived recovery (patient-reported recovery rate, symptom count, fatigue visual analogue scale (VAS), breathlessness VAS), physical health status (dyspnoea-12 score [19], Functional Assessment of Chronic Illness Therapy fatigue score [20], Washington Group Short Set on Functioning score [21], incremental shuttle walk test distance [22], Short Physical Performance Battery score [23]), cognitive impairment and mental health status (Montreal Cognitive Assessment) score) [24], Generalised Anxiety Disorder-7 score [25], Patient Health Questionnaire-8 score [26], Post-traumatic Stress Disorder Checklist-5 score [27]) and organ function (forced expiratory volume in 1 s (FEV_1_), forced vital capacity (FVC), FEV_1_/FVC ratio, carbon monoxide transfer coefficient, transfer factor of the lung for carbon monoxide, brain-natriuretic peptide, haemoglobin A1C, estimated glomerular filtration rate, C-reactive protein, fibrinogen).

### Bias

Several potential sources of bias were considered *a priori*, as follows: 1) bias in treatment decisions made by clinicians (prior to corticosteroids becoming standard care in June 2020); 2) selection bias regarding who participated in the PHOSP-COVID study; and 3) survivor bias due to participants being recruited to PHOSP-COVID after hospital discharge (*i.e.*, survivors). A statistical analysis plan was developed including the use of propensity weighting to ensure balance between treatment groups in the primary analysis and sensitivity analyses using data from the ISARIC study.

### Statistical analysis

The main analysis was undertaken using the PHOSP-COVID cohort. A logistic regression model was fitted to estimate propensity for exposure to corticosteroids. An average treatment effect of corticosteroid treatment on the outcomes (primary and secondary) was calculated weighted by the inverse of propensity for exposure using either linear or logistic regression, depending on the distribution of the outcome. The following variables, which potentially influence treatment decisions, were included in the propensity model: age, sex, obesity status, ethnicity, index of multiple deprivation [28], WHO Clinical Progression Scale status, smoking status, presence of specific comorbidities (cardiovascular, respiratory, metabolic/endocrine/renal, neurological/psychiatric (defined in table S1)) and total number of comorbidities. Multiple Imputation by Chained Equations was performed to deal with missing data for the variables used in the propensity model. Summary statistics tables were produced for patients by exposure status, visually inspecting the distribution of propensity scores and evaluating imbalance between groups by standardised mean difference (SMD).

### Sensitivity analyses

Sensitivity analyses were performed using the ISARIC dataset to address selection, treatment and survivor biases in PHOSP-COVID (supplementary methods). In summary, a propensity score weighting for corticosteroid treatment was developed in the ISARIC cohort (survivors and nonsurvivors) using logistic regression. The PHOSP-COVID dataset was used to develop a prediction model for EQ-5D-5L UI at 1 year. We used this model to calculate predicted 1-year EQ-5D-5L UI values for those that survived COVID-19 hospitalisation in the ISARIC cohort (1000 estimates per patient). Adults that did not survive were assigned an EQ-5D-5L UI value of zero. Participants who were in both ISARIC and PHOSP-COVID cohorts were assigned their PHOSP-COVID EQ-5D-5L UI value. The 1000 datasets created were sub-sampled down to the PHOSP-COVID dataset size to ensure robust standard errors (1000 random samples of each dataset). These datasets were used to produce an average treatment effect of corticosteroid exposure on EQ-5D-5L UI weighted by the inverse of propensity for exposure using linear regression.

The sensitivity analysis addressed selection and survivor bias by using the structure of the ISARIC population (assuming the ISARIC population was similar to all hospitalised patients with COVID-19 eligibility to receive corticosteroids). The ISARIC cohort included participants who did not survive hospitalisation with COVID-19. Biased treatment assignment was accounted for by developing a propensity score with corticosteroid as the dependent variable, which was developed in the ISARIC cohort and therefore independent of survival status at hospital discharge.

Statistical analysis was undertaken using R (version 4.2.0) with the *tidyverse, tidymodels, mice, finalfit, WeightIt* and *tableone* packages for all statistical analyses. The study is reported using the Strengthening the Reporting of Observational Studies in Epidemiology (STROBE) reporting guidelines.

### Permissions

PHOSP-COVID was approved by the Leeds West Research Ethics Committee (20/YH/0225) and is registered on the ISRCTN Registry (ISRCTN10980107). ISARIC was approved by the South Central – Oxford C Research Ethics Committee in England and the Scotland A Research Ethics Committee.

## Results

The relevant PHOSP-COVID cohort consisted of 2697 participants, of whom 2248 required at least supplemental oxygen and were discharged from hospital between 1 February 2020 and 31 March 2021. There were 1888 participants with nonmissing corticosteroid information not prescribed immunosuppressant medication pre-hospital, of which 1149 (60.9%) were corticosteroid-exposed and 739 (39.1%) were corticosteroid-nonexposed. 1226 participants had an EQ-5D-5L UI score at their 1-year visit and 1057 participants had both pre-hospital and 1-year EQ-5D-5L UI scores (figure 1). There were no meaningful differences in baseline characteristics between included participants and those excluded due to absent 1-year EQ-5D-5L UI data (table S2).

Baseline characteristics for the 1888 included participants demonstrated a mean age of 58.6 years with 64.4% being male. 75.1% were white, 10.1% South Asian, 7.3% black and 7.5% other ethnicity. 58.6% were obese (body mass index ≥30 kg·m^−2^), and 43.8% had two or more comorbidities (table S3). Prior to propensity weighting some baseline characteristics were imbalanced between treatment groups, as demonstrated by an SMD of >0.1 (table S3). Participants treated with corticosteroids were slightly younger compared to those not receiving corticosteroids (58.0 *versus* 59.7 years) and had greater prevalence of white ethnicity (76.8% *versus* 72.5%), deprivation (49.5% *versus* 41.0% in lowest two deprivation index quintiles) and obesity (61.0% *versus* 55.8%). The corticosteroid group had a lower proportion of “never-smokers” (54.9% *versus* 56.4%). There were also differences in the level of respiratory support required between patients treated with corticosteroid and those not: 51.5% *versus* 54.7% received low-flow oxygen (WHO scale 5), 33.3% *versus* 22.6% received noninvasive respiratory support (WHO scale 6) and 15.1% *versus* 22.7% received invasive mechanical ventilation (WHO scale 7–9).

Propensity weighting successfully achieved balance between the treatment groups, as demonstrated by an SMD <0.1 for all recorded baseline outcomes (table 1).

**TABLE 1:** Baseline characteristics after propensity weighting

Characteristic		Corticosteroids	No corticosteroids	SMD
**Subjects**		1147.93	740.15	
**Age at admission, years**		58.52±11.89	58.50±12.60	0.002
**Sex**	Male	741.6 (64.6)	479.2 (64.7)	0.003
	Female	406.3 (35.4)	261.0 (35.3)	
**Ethnicity**	White	862.8 (75.2)	558.3 (75.4)	0.008
	South Asian	118.2 (10.3)	76.2 (10.3)	
	Black	83.5 (7.3)	53.4 (7.2)	
	Other	83.4 (7.3)	52.3 (7.1)	
**IMD quintile**	1 – most deprived	262.4 (22.9)	169.1 (22.9)	0.008
	2	269.6 (23.5)	172.6 (23.3)	
	3	202.5 (17.6)	132.8 (17.9)	
	4	196.2 (17.1)	126.6 (17.1)	
	5 – least deprived	217.1 (18.9)	139.0 (18.8)	
**Obesity**	Yes – BMI ≥30 kg·m^−2^	683.3 (59.5)	440.1 (59.5)	0.001
	No – BMI <30 kg·m^−2^	464.6 (40.5)	300.0 (40.5)	
**Smoking status**	Never	642.7 (56.0)	412.0 (55.7)	0.007
	Ex-smoker	484.6 (42.2)	314.3 (42.5)	
	Current smoker	20.7 (1.8)	13.8 (1.9)	
**Number of comorbidities**		1.48±1.37	1.49±1.40	0.005
**Number of comorbidities**	No comorbidity	342.2 (29.8)	222.1 (30.0)	0.013
	1 comorbidity	308.5 (26.9)	202.2 (27.3)	
	2+ comorbidities	497.2 (43.3)	315.8 (42.7)	
**Cardiovascular comorbidities**	Yes	562.6 (49.0)	361.6 (48.9)	0.003
	No	585.4 (51.0)	378.6 (51.1)	
**Metabolic/endocrine/renal comorbidities**	Yes	314.7 (27.4)	198.8 (26.9)	0.012
	No	833.2 (72.6)	541.4 (73.1)	
**Respiratory comorbidities**	Yes	292.3 (25.5)	190.1 (25.7)	0.005
	No	855.6 (74.5)	550.1 (74.3)	
**Type 2 diabetes**	Yes	238.5 (20.8)	151.4 (20.5)	0.008
	No	909.4 (79.2)	588.8 (79.5)	
**Neurological/psychiatric comorbidities**	Yes	52.1 (4.5)	31.8 (4.3)	0.012
	No	1095.8 (95.5)	708.4 (95.7)	
**WHO clinical progression scale status**	WHO scale 5	603.3 (52.6)	388.1 (52.4)	0.002
	WHO scale 6	335.0 (29.2)	216.3 (29.2)	
	WHO scale 7–9	209.6 (18.3)	135.7 (18.3)	

Data are n, n (%) or mean±sd. Percentages are calculated by category after exclusion of missing data for that variable. BMI: body mass index; IMD: index of multiple deprivation; SMD: standardised mean difference; WHO: World Health Organization.

### Primary outcomes

**FIGURE 2:**
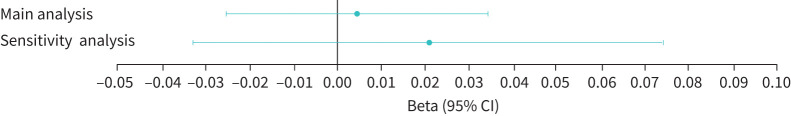
EuroQol-Five Dimensions–Five Levels utility index at 1 year after hospital discharge in corticosteroid exposed *versus* nonexposed patients. Between-group mean difference and 95% confidence interval shown for main analysis (Post-hospitalisation coronavirus disease 2019 (PHOSP-COVID) cohort) and sensitivity analysis (International Severe Acute Respiratory and emerging Infection Consortium (ISARIC) cohort).

**FIGURE 3:**
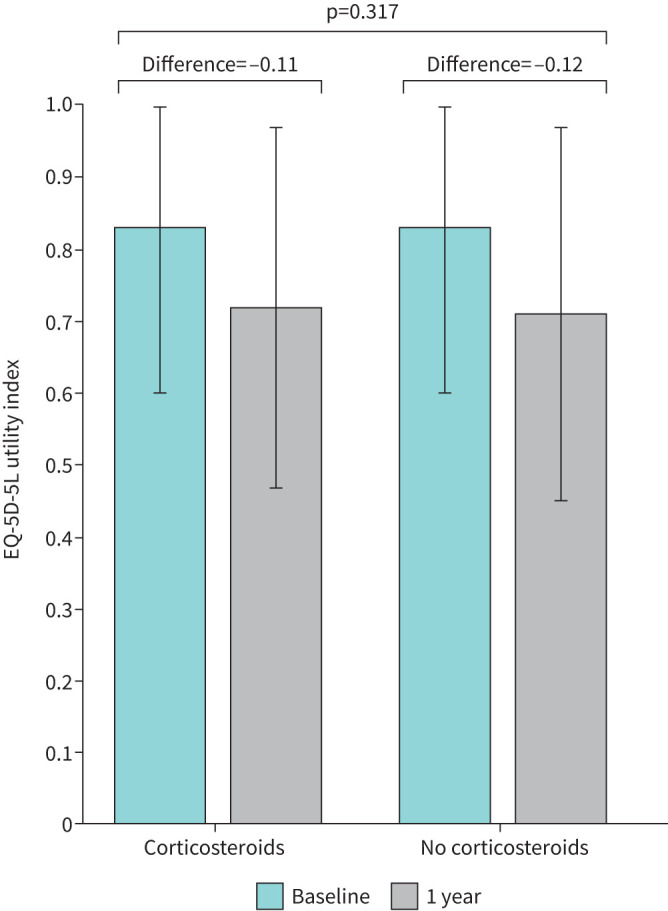
EuroQol-Five Dimensions–Five Levels (EQ-5D-5L) utility index change from pre-hospital (baseline) to 1 year in corticosteroid-exposed *versus* nonexposed patients.

After propensity weighting for treatment, there was no statistically significant difference in EQ-5D-5L UI at 1 year between corticosteroid exposed (mean±sd 0.72±0.25) and nonexposed (0.71±0.25) groups (mean difference 0.004, 95% CI −0.026–0.034, p=0.77) (table 2 and figures 2 and 3).

**TABLE 2:** Primary and secondary outcomes: patient-reported outcomes, mental health status and cognitive assessments

Outcome		Corticosteroids	No corticosteroids	p-value
**Subjects**		737.5	488.9	
**EQ-5D-5L UI at 1 year**		0.72±0.25	0.71±0.25	0.773
**EQ-5D-5L UI change pre-hospital to 1 year**		−0.11±0.22	−0.12±0.22	0.317
**Do you feel fully recovered from COVID-19?**	Yes	223.1 (30.2)	139.1 (28.5)	0.811
	No/not sure	465.3 (63.1)	299.6 (61.3)	
	Missing data	49.2 (6.7)	50.2 (10.3)	
**Any symptom at 1 year**	Yes	656.8 (89.1)	423.6 (86.6)	0.508
	No	39.9 (5.4)	21.4 (4.4)	
	Missing data	40.8 (5.5)	43.9 (9.0)	
**Symptom count**		8.00 (4.00–16.00)	9.00 (4.00–16.00)	0.671
**Fatigue VAS**		2.00 (0.00–5.00)	3.00 (0.00–5.00)	0.465
**Breathlessness VAS**		1.00 (0.00–4.00)	1.00 (0.00–5.00)	0.043
**Dyspnoea-12 score**		5.04±7.22	5.46±7.83	0.373
**FACIT fatigue score**		36.79±12.23	36.31±12.87	0.524
**MoCA corrected**		26.90±3.22	26.65±3.23	0.232
**MoCA corrected <23**	Yes	49.6 (6.7)	41.7 (8.5)	0.373
	No	516.8 (70.1)	356.6 (72.9)	
	Missing data	171.1 (23.2)	90.6 (18.5)	
**WG-SS score**		2.00 (0.00–4.00)	2.00 (0.00–4.00)	0.613
**GAD-7 total score**		4.75±5.46	4.91±5.60	0.631
**Anxiety (GAD-7 score >8)**	Yes	159.6 (21.6)	110.5 (22.6)	0.684
	No	576.0 (78.1)	375.8 (76.9)	
	Missing data	<5	<5	
**PHQ-8 total score**		6.14±6.25	6.21±6.39	0.791
**PCL-5 total score**		13.63±16.76	13.76±17.54	0.901
**PTSD (PCL-5 score ≥38)**	Yes	79.6 (10.8)	51.0 (10.4)	0.866
	No	650.8 (88.2)	431.1 (88.2)	
	Missing data	7.1 (1.0)	6.7 (1.4)	

Data are n, n (%), mean±sd or median (interquartile range). Percentages are calculated by category after exclusion of missing data for that variable. COVID-19: coronavirus disease 2019; EQ-5D-5L UI: EuroQol-Five Dimensions–Five Levels utility index; FACIT: Functional Assessment of Chronic Illness Therapy; GAD-7: Generalized Anxiety Disorder seven-item; MoCA: Montreal Cognitive Assessment; PCL-5: Post-Traumatic Stress Disorder Checklist for the Diagnostic and Statistical Manual of Mental Disorders; PHQ-8: Patient Health Questionnaire-8; PTSD: post-traumatic stress disorder; VAS: visual analogue scale; WG-SSL Washington Group Short Set.

There was a large reduction in EQ-5D-5L UI from pre-hospital to 1 year, with no significant difference between corticosteroid exposed (mean change −0.12 (0.22)) and nonexposed (−0.11 (0.22)) groups (mean difference 0.01, 95% CI −0.01–0.04, p=0.32) (table 2 and figure 3).

### Secondary outcomes

**FIGURE 4:**
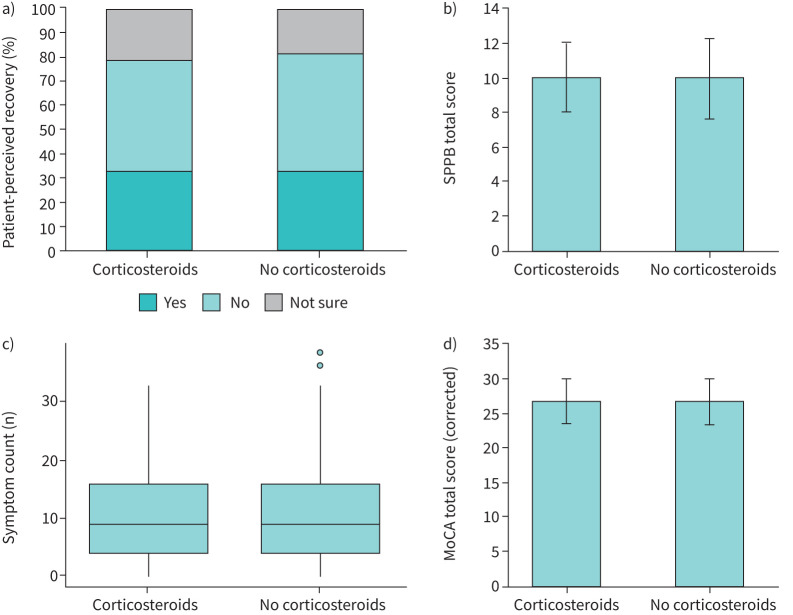
Secondary outcomes. a) Patient-perceived recovery, b) Short Physical Performance Battery (SPPB) score, c) symptom count and d) Montreal Cognitive Assessment (MoCA) score (corrected) 1 year after hospital discharge in corticosteroid exposed *versus* nonexposed patients.

Secondary outcomes, assessing patient-reported outcomes, physical, cognitive and mental health status, and measurements of organ impairment, were not significantly different between treatment groups at 1 year (tables 2 and 3, and figure 4), except breathlessness VAS, which was lower in patients who had received corticosteroids (median (interquartile range) 1.0 (0.0–4.0) *versus* 1.0 (0.0–5.0), p=0.043).

**TABLE 3:** Secondary outcomes: physical impairment and organ function

Outcome		Corticosteroids	No corticosteroids	p-value
**Subjects**		737.5	488.9	
**ISWT distance, m**		462.78±468.30	455.15±252.13	0.770
**ISWT % pred**		62.59±59.25	60.59±28.72	0.604
**SPPB total score**		10.12±1.99	9.93±2.32	0.160
**SPPB (mobility disability ≤10)**	Yes	289.4 (39.2)	219.0 (44.8)	0.671
	No	325.1 (44.1)	233.1 (47.7)	
	Missing	122.9 (16.7)	36.8 (7.5)	
**FEV_1_ % pred <80%**	Yes	78.1 (10.6)	70.3 (14.4)	0.613
	No	258.1 (35.0)	210.6 (43.1)	
	Missing data	401.3 (54.4)	208.0 (42.5)	
**FVC % pred <80%**	Yes	85.1 (11.5)	74.3 (15.2)	0.772
	No	251.1 (34.0)	207.3 (42.4)	
	Missing data	401.3 (54.4)	207.3 (42.4)	
**FEV_1_/FVC <0.7**	Yes	32.5 (4.4)	31.1 (6.4)	0.606
	No	310.6 (42.1)	257.8 (52.7)	
	Missing data	394.4 (53.5)	200.0 (40.9)	
***K*_CO_ <80% pred**	Yes	15.6 (2.1)	9.3 (1.9)	0.287
	No	103.6 (14.0)	98.0 (20.1)	
	Missing data	618.3 (83.8)	381.6 (78.0)	
***T*_LCO_ <80% pred**	Yes	19.2 (2.6)	27.7 (5.7)	0.074
	No	92.4 (12.5)	71.4 (14.6)	
	Missing data	625.8 (84.9)	389.8 (79.7)	
**BNP ≥100 ng·L^−1^ or NT-proBNP ≥400 ng·L^−1^**	Yes	23 (3.2)	24 (4.9)	0.529
	No	292 (39.9)	229 (46.3)	
	Missing data	416 (56.9)	242 (48.9)	
**HbA1C ≥6.0% (DCCT/NGSP)**	Yes	157 (21.5)	114 (23.0)	0.881
	No	277 (37.9)	196 (39.6)	
	Missing data	297 (40.6)	185 (37.4)	
**eGFR <60 mL·min^−1^ per 1.73 m^2^**	Yes	74 (10.1)	63 (12.7)	0.586
	No	475 (65.0)	321 (64.8)	
	Missing data	182 (24.9)	111 (22.4)	
**C-reactive protein concentration >5 mg·L^−1^**	Yes	124 (17.0)	78 (15.8)	0.204
	No	423 (57.9)	321 (64.8)	
	Missing data	184 (25.2)	96 (19.4)	
**Fibrinogen (g·L^−1^)**		3.58±2.23	3.56±0.87	0.846

Data are n, n (%) or mean±sd. Percentages are calculated by category after exclusion of missing data for that variable. BNP: brain natriuretic peptide; DCCT: Diabetes Control and Complications Trial; eGFR: estimated glomerular filtration rate; FEV_1_: forced expiratory volume in 1 s; FVC: forced vital capacity; HbA1C: haemoglobin A1C; ISWT: incremental shuttle walk test; *K*_CO_: carbon monoxide transfer coefficient; NGSP: National Glycohemoglobin Standardization Program; NT-proBNP: N-terminal pro-brain natriuretic peptide; SPPB: Short Physical Performance Battery; *T*_LCO_: transfer factor of the lung for carbon monoxide.

### Sensitivity analysis

In the sensitivity analysis, there was no significant difference in the EQ-5D-5L UI at 1 year between patients who received corticosteroids and those who did not (between-group difference 0.021, 95% CI −0.033–0.074, p=0.45) (figure 2).

## Discussion

To our knowledge, this is the first report investigating the effect of acute corticosteroids on HRQoL, other patient-reported outcomes, physical and mental health status, and multi-system organ effects 1 year after hospitalisation for COVID-19. We observed large reductions in HRQoL at 1 year and report novel findings that there was neither a difference in EQ-5D-5L UI at 1 year, nor in EQ-5D-5L UI change pre-hospital to 1 year, between patients who did or did not receive corticosteroids for their acute illness. There remained no difference in HRQoL at 1 year after adjusting for survivor and selection bias using a large cohort of patients admitted with COVID-19 (ISARIC cohort). We also found no difference between receipt of acute corticosteroids or not across a range of secondary end-points assessing patient-reported outcomes, physical, cognitive and mental health status, and measurements of multi-system organ impairment. Despite the observational longitudinal nature of our study, it is likely to be the most comprehensive and robust data available, as the large acute randomised controlled trials of therapeutics in COVID-19 were unable to perform in-person follow-up assessments [1–4] and corticosteroids are now standard of care for COVID-19 requiring supplemental oxygen, meaning a placebo-controlled trial would now be unethical [17]. Our recruitment period encompassed time before and after systemic corticosteroids became standard care for patients requiring oxygen due to COVID-19 (June 2020), allowing comparison between corticosteroid exposed and nonexposed groups.

Our data demonstrate the significant negative impact on HRQoL and other health outcomes 1 year after hospital discharge in this population, similar to our previous reports but in a larger sub-set [6]. Pre-hospital our cohort reported EQ-5D-5L UI scores in line with normal values (reported as 0.81 for men and 0.79 for women aged 55–59 years) [29]. 1 year after discharge from hospital, the EQ-5D-5L UI was comparable to long-term health conditions such as COPD [30].

Developments in treatments for acute COVID-19 (including pharmacological therapies, such as corticosteroids, and ventilation strategies), combined with effective vaccines, have significantly reduced the risk of in-hospital COVID-19 mortality. However, the risk of long COVID remains, and although risk increases with more severe acute illness [5], many people with mild acute COVID-19 develop persistent health problems. We have previously shown that elevated inflammatory proteins 5 months after COVID-19 hospital discharge are associated with increased risk of very severe health impairments at 1 year [5]; therefore, it was reasonable to hypothesise that the anti-inflammatory effect of corticosteroids could mitigate the risk of long COVID. A previous study found no difference in HRQoL 180 days after hospital discharge from a higher 12 mg dose of dexamethasone compared to the standard 6 mg dose [31], but HRQoL comparisons between corticosteroid treated or not were not available. Another recent study showed a reduction in the duration of post-COVID-19 symptoms reported by patients who had received dexamethasone, compared to those who did not [32]. This was not consistent with our own data, which showed no significant difference in presence of any symptoms, or the number of symptoms, at 1 year.

Other acute pharmacological interventions have shown promising effects on the risk of long COVID. Anti-IL-6 (tocilizumab) improves HRQoL at 6 months in COVID-19 survivors admitted to intensive care [13], although whether this benefit applies to patients outside of intensive care is unknown. The antiviral remdesivir is associated with a reduction in rates of long COVID at 180 days, although the study excluded severely unwell patients so this benefit may not apply to a broader population [33]. The antivirals nirmatrelvir and molnupiravir both reduce the risk of post-acute sequelae of COVID-19, including fatigue, muscle pain and neurocognitive impairment at 180 days [34, 35]. *Post hoc* analysis of nebulised interferon-beta-1a for COVID-19 showed reductions in fatigue/malaise and loss of taste or smell at 60–90 days compared to placebo and further investigations are ongoing [36]. Metformin reduces the risk of long COVID in nonhospitalised overweight and obese patients, although the effect in more severe disease is unknown [37]. While the results of these trials are encouraging, it is noteworthy that each has limitations to their applicability in a wider patient population and none have provided strong enough evidence to change treatment guidelines with the aim of reducing long COVID. The HEAL-COVID study reported no benefit from 2 weeks of anticoagulation (apixaban) on post-discharge mortality or hospital readmission but has not yet reported quality of life outcomes [38]. A second study arm investigating 12 months of atorvastatin is underway [39].

Trials of potential treatments for patients with persistent health problems beyond the acute COVID-19 illness are being undertaken, although are few in number. In a phase 2 placebo-controlled trial, 4 weeks of AXA1125 (an endogenous metabolic modulator comprising five amino acids and N-acetylcysteine) improved fatigue scores in patients with persistent fatigue at least 12 weeks after COVID-19 [40]. The STIMULATE-ICP (Symptoms, Trajectory, Inequalities and Management: Understanding Long-COVID to Address and Transform Existing Integrated Care Pathways) study will investigate the effect of antihistamine (famotidine/loratidine), anticoagulation (rivaroxaban) and anti-inflammatory (colchicine) medications on long COVID recovery, in addition to interventions such as rehabilitation strategies [41]. The PHOSP-I study will investigate tocilizumab in patients with persistent symptoms at least 3 months after COVID-19 and evidence of persistent systemic inflammation [42]. Given the evidence for acute interventions not reducing long COVID across a broad patient population, these trials and others are urgently needed to reduce post-COVID-19 sequelae including long COVID. Additionally, although COVID-19 vaccination prior to infection reduces the risk of developing long COVID, it does not appear to improve long COVID in those already affected [43].

Our study has a number of strengths. We included a large cohort of patients discharged from hospital after receiving oxygen for COVID-19 and our sensitivity analysis uses ISARIC data to verify our findings in a much larger hospitalised cohort also requiring oxygen. Therefore, we are confident that our findings are applicable to patients meeting guideline criteria for corticosteroid treatment for COVID-19. Additionally, we used propensity weighting to ensure balance between groups prior to analysing 1-year outcomes in an attempt to replicate the effect of randomised allocation and account for elements of biasing. We are confident, therefore, that the lack of benefit from acute corticosteroids observed here is genuine.

Our study has some limitations. First, despite using propensity-weighting methods, this is an observational study and therefore unable to fully replicate a randomised trial. Our statistical methods were designed to minimise potential biases related to this, but some residual effect may remain. Second, we included patients admitted to hospital over a 14-month period, spanning waves of different COVID-19 variants and the early stages of the vaccine rollout. We cannot exclude potential effects due to these factors, particularly as our corticosteroid nonexposed participants were predominantly hospitalised before June 2020 and corticosteroid-exposed participants predominantly after June 2020. Third, the PHOSP-COVID cohort had a more severe acute illness than the general hospitalised COVID-19 population and only includes patients who survived at least 5 months after discharge; it is therefore subject to selection and survivor biases. We have attempted to address these in our sensitivity analysis, using the ISARIC cohort which includes patients who died. Fourth, there is a significant amount of missing lung function data due to variable infection prevention restrictions during the study period. Therefore, we cannot fully exclude a possible effect on lung function. Finally, pre-hospital EQ-5D-5 L UI was assessed retrospectively using patient recollection of their quality of life prior to hospitalisation with COVID-19. These data are therefore subject to recall bias, although the effect is likely equal between the treatment groups.

There remains a large reduction in HRQoL and other health outcomes 1 year after hospitalisation for COVID-19. Studies to identify pharmacological and nonpharmacological interventions given after the acute COVID-19 illness are essential to address this. It is also important to seek better mechanistic understanding of post-COVID-19 sequelae and improve phenotyping of patients who may respond to specific interventions.

In conclusion, we found no long-term benefit on HRQoL or other health outcomes from corticosteroids given to treat acute COVID-19. There remains an urgent need for effective interventions that reduce the long-term burden of health issues following COVID-19.
